# Editorial: Advancing biomechanics: enhancing sports performance, mitigating injury risks, and optimizing athlete rehabilitation

**DOI:** 10.3389/fspor.2025.1556024

**Published:** 2025-02-04

**Authors:** Wissem Dhahbi

**Affiliations:** ^1^ Research Unit “Sport Sciences, Health and Movement”, High Institute of Sports and Physical Education of Kef, University of Jendouba, Kef, Tunisia; ^2^ Qatar Police Academy, Training Department, Police College, Doha, Qatar

**Keywords:** computer-assisted movement analysis, electrophysiological monitoring, kinematic data collection, motion sensing technology, neuromuscular assessment, performance metrics, signal processing, wearable technology

**Editorial on the Research Topic**
Advancing biomechanics: enhancing sports performance, mitigating injury risks, and optimizing athlete rehabilitation

## Introduction

The field of biomechanics has undergone a transformative evolution, driven by rapid advancements in both hardware and software technologies. These innovations have not only enhanced our understanding of human movement but have also bridged the gap between research and practical applications in sports medicine, performance optimization, and injury rehabilitation. This special issue, titled *Advancing Biomechanics: Enhancing Sports Performance, Mitigating Injury Risks, and Optimizing Athlete Rehabilitation*, aims to showcase the latest research and technological developments that are shaping the future of biomechanics in sports. The contributing articles in this special issue highlight the diverse applications of biomechanics, ranging from the analysis of sports techniques to the development of novel rehabilitation strategies. Each study underscores the importance of evidence-based approaches in addressing the complex challenges faced by athletes and sports practitioners. In this editorial, we will summarize the key findings of these studies, discuss their implications, and place them within the broader context of biomechanics research.

## Enhancing sports performance through biomechanical analysis

One of the primary goals of biomechanics is to optimize athletic performance by analyzing and refining movement patterns. The study by Trigt et al. provides a compelling example of how biomechanical analysis can enhance performance in elite athletes. By using inertial measurement units (IMUs), the researchers were able to capture detailed kinematic data during tennis serves. Their findings revealed that while the kinetic chain principle is generally followed, professional players often deviate from the proximal-to-distal sequence, particularly in second serves. This study highlights the importance of segmental angular velocities, especially in the trunk and upper arm, in achieving high ball speeds. These insights can inform coaching strategies and training programs aimed at improving serve performance.

Similarly, the study Skujyte et al. demonstrates how innovative technologies can be used to assess and enhance sprint performance in young football players. The Alex7 device, which provides both resisted and assisted sprinting conditions, was found to be highly reliable for measuring kinematic variables such as stride length and ground contact time. Although the device tended to overestimate running times, its ability to create controlled training environments offers valuable opportunities for performance optimization. These findings underscore the potential of technology-driven training solutions in improving athletic performance across various sports.

## Mitigating injury risks through biomechanical interventions

Several contributions advanced our understanding of injury mechanisms and prevention strategies. The study Wilkerson et al. explores the relationship between concussion history and subsequent musculoskeletal injuries. The finding that female athletes with multiple previous concussions showed elevated risk for core and lower extremity injuries highlights the complex interplay between neurological and musculoskeletal health. This study highlights the potential of virtual-reality as a tool for assessing perceptual-motor impairments and developing targeted interventions to reduce injury risk.

Another important contribution to injury prevention comes from the study Field et al. This research validates the use of instrumented-mouthguards for detecting direct head impacts in rugby players. The instrumented-mouthguard demonstrated high sensitivity and positive predictive value, making it a valuable tool for monitoring head impacts during games. By identifying high-risk scenarios, such as impacts during ruck contests, this technology can inform strategies for reducing the incidence of head injuries in contact sports.

## Optimizing athlete rehabilitation through biomechanics-based approaches

Rehabilitation is a crucial component of athlete care, and biomechanics offers innovative approaches to optimize recovery and prevent re-injury. The study Issaoui et al. provides valuable insights into the postoperative recovery of ACL-reconstructed patients. The researchers compared the effects of different autografts on quadriceps and hamstring strength recovery, finding that the hamstring-tendon graft offered the most balanced recovery in terms of muscle strength and knee stability. These findings can guide clinicians in selecting the most appropriate graft type for individual patients, thereby improving rehabilitation outcomes.

In addition, the systematic review Sanchez-Alvarado et al. evaluates the efficacy of various conservative treatments for iliotibial-band-syndrome. The review highlights the effectiveness of hip abductor strengthening exercises, particularly when combined with shockwave or manual therapy, in reducing pain and improving function in runners. This study emphasizes the importance of evidence-based rehabilitation strategies in managing common running injuries and facilitating a safe return to sport.

## Emerging technologies and methodologies in biomechanics

The integration of advanced technologies into biomechanics research has opened new avenues for understanding human movement and developing innovative solutions for sports performance and injury management. The study Philipp et al. demonstrates the reliability of markerless motion capture (MMC) systems in assessing human movement. The researchers found that MMC systems exhibit good to excellent reliability for capturing kinematic variables, with biological variability being the primary source of error. These findings support the use of MMC systems as a valid and practical tool for movement analysis in both research and clinical settings.

Another notable contribution is the pilot study Paksoy et al. which explores the variability in force profiles during fatiguing tasks. The study reveals significant inter-individual differences in fatigue characteristics, which may be influenced by physiological, technical, and motivational factors. These findings have important implications for understanding spinal stability and developing personalized training programs to reduce the risk of injury during fatiguing activities.

## Conclusion and future directions

The studies featured in this special issue collectively highlight the transformative impact of biomechanics on sports performance, injury prevention, and athlete rehabilitation. By leveraging cutting-edge technologies and evidence-based approaches, researchers and practitioners can address the complex challenges faced by athletes and optimize their performance and well-being. However, several areas warrant further investigation. For example, the integration of machine learning and artificial intelligence into biomechanical analysis holds promise for enhancing the accuracy and efficiency of movement assessments. Additionally, longitudinal studies are needed to evaluate the long-term effects of biomechanical interventions on injury prevention and rehabilitation outcomes.

